# Development of an indirect ELISA, blocking ELISA, fluorescent microsphere immunoassay and fluorescent focus neutralization assay for serologic evaluation of exposure to North American strains of Porcine Epidemic Diarrhea Virus

**DOI:** 10.1186/s12917-015-0500-z

**Published:** 2015-08-01

**Authors:** Faten Okda, Xiaodong Liu, Aaron Singrey, Travis Clement, Julie Nelson, Jane Christopher-Hennings, Eric A. Nelson, Steven Lawson

**Affiliations:** Veterinary & Biomedical Sciences Department, South Dakota State University, Brookings, SD USA; National Research Center, Giza, Egypt

**Keywords:** Porcine epidemic diarrhea virus (PEDV), Serology, ELISA, Fluorescent microsphere immunoassay (FMIA), Fluorescent Focus Neutralization (FFN)

## Abstract

**Background:**

Recent, severe outbreaks of porcine epidemic diarrhea virus (PEDV) in Asia and North America highlight the need for well-validated diagnostic tests for the identification of PEDV infected animals and evaluation of their immune status to this virus. PEDV was first detected in the U.S. in May 2013 and spread rapidly across the country. Some serological assays for PEDV have been previously described, but few were readily available in the U.S. Several U.S. laboratories quickly developed indirect fluorescent antibody (IFA) assays for the detection of antibodies to PEDV in swine serum, indicating prior exposure. However, the IFA has several disadvantages, including low throughput and relatively subjective interpretation. Different serologic test formats have advantages and disadvantages, depending on the questions being asked, so a full repertoire of tests is useful. Therefore, the objective of this study was to develop and validate multiple improved serological assays for PEDV, including an indirect ELISA (iELISA); a highly specific monoclonal antibody-based blocking ELISA (bELISA); fluorescent microsphere immunoassays (FMIA) that can be multiplexed to monitor exposure to multiple antigens and pathogens simultaneously; and a fluorescent focus neutralization assay (FFN) to measure functional virus neutralizing antibodies.

**Results:**

A recombinant North American nucleoprotein (NP) based iELISA was developed and validated along with a bELISA using newly developed PEDV-NP specific biotinylated monoclonal antibodies (mAbs) and an FMIA using magnetic beads coupled with expressed NA PEDV-NP. Receiver operating characteristic (ROC) analysis was performed using swine serum samples (iELISA n = 1486, bELISA n = 1186, FMIA n = 1420). The ROC analysis for the FMIA showed estimated sensitivity and specificity of 98.2 and 99.2 %, respectively. The iELISA and bELISA showed a sensitivity and specificity of 97.9 and 97.6 %; and 98.2 and 98.9 %, respectively. Inter-rater (kappa) agreement was calculated to be 0.941 between iELISA and IFA, 0.945 between bELISA and IFA and 0.932 between FMIA and IFA. Similar comparative kappa values were observed between the iELISA, bELISA and FMIA, which demonstrated a significant level of testing agreement among the three assays. No cross-reactivity with the closely related coronaviruses, transmissible gastroenteritis virus (TGEV) or porcine respiratory coronavirus (PRCV) was noted with these assays. All three assays detected seroconversion of naïve animals within 6–9 days post exposure. The FFN assay allows relative quantitation of functional neutralizing antibodies in serum, milk or colostrum samples.

**Conclusion:**

Well-validated iELISA, bELISA and FMIA assays for the detection of PEDV antibodies were developed and showed good correlation with IFA and each other. Each assay format has advantages that dictate how they will be used in the field. Newly developed mAbs to the PEDV-NP were used in the bELISA and for expediting FFN testing in the detection and quantitation of neutralizing antibodies. In addition, these PEDV mAbs are useful for immunohistochemistry, fluorescent antibody staining and other antigen-based tests. Measurement of neutralizing antibody responses using the FFN assay may provide a valuable tool for assessment of vaccine candidates or protective immunity.

## Background

Porcine epidemic diarrhea virus (PEDV) was first described in Europe in the 1970s with more recent and severe outbreaks in Asia [1, 2]. The virus was identified in the United States in May 2013, causing severe diarrhea and vomiting in pigs across age groups, with high mortality of up to 90−95 % in suckling pigs [3]. PEDV is an enveloped, single stranded RNA virus belonging to the *Coronaviridae* family. The coronaviruses taxonomically form a subfamily (*Coronavirinae*) within the order *Nidovirales*. Recently, the International Committee on Taxonomy of Viruses (ICTV) recognized four genera within the *Coronavirinae* subfamily: *Alphacoronavirus*, *Betacoronavirus*, *Gammacoronavirus*, and *Deltacoronavirus* [4]. PEDV belongs to the genus *Alphacoronavirus* along with other swine viruses including transmissible gastroenteritis virus (TGEV) and porcine respiratory coronavirus (PRCV).

The genome is composed of a large ~28 Kb molecule consisting of a 5′ untranslated region (UTR), a 3′ UTR, and at least seven open reading frames (ORFs) encoding three nonstructural proteins: ORF1ab (pp1a and pp1ab) and ORF3, an accessory protein. The four major structural proteins of the mature virion include the spike (S) glycoprotein (Mr 150–220 kDa), the nucleoprotein (NP) (Mr 45–57 kDa) that is associated with the positive stranded RNA providing integral support for its helical structure, the glycosylated membrane protein (M) (Mr 20–30 kDa), and the glycosylated envelope protein (E) (Mr 7 kDa) [5–7].

Coronaviruses are taxonomically assigned to different genera based on their rooted phylogeny and calculated evolutionary distance for seven highly conserved genomic domains within ORF 1ab [8]. The genetic diversity of coronaviruses may be due to their high frequency of recombination [9]. The heterogeneity among coronavirus subfamilies is well documented [7], and the factors that contribute to PEDV’s ability to gain or lose parts of its transcriptome are believed to have contributed to quasispecies with novel traits that are able to adapt to new hosts, ecological niches and zoonotic events. The exact origin of PEDV in North America is not entirely clear, but there is evidence of genetic similarities to Chinese PEDV strains [10].

Recently, a novel NA PEDV recombinant strain was identified (S INDEL) containing both insertions and deletions within the N-terminal domain of the ORF 3 and S1 genes. Specifically, sequence alignment indicated spike gene nucleotide deletions at positions 164–169 that correspond to amino acid deletions at positions 55 and 56 in addition to substitutions at positions 23 (I), 31 (H), 57 (K), and 59 (E) as compared to the CV777strain [10, 11].

The relatedness of several PEDV strains circulating in China was evaluated by Li et al. [5] using phylogenic analysis of the NP gene and no insertions or deletions were noted. Sequence comparison with other European and Korean PEDV strains obtained from GenBank indicated that the NP genes were highly conserved (94.7−97.7 %) even though these strains originated from different geographic regions [5]. In addition to being highly conserved among PEDV variants, the NP is the most abundant viral protein expressed in PEDV infected cells [12, 13]. In contrast, the spike protein is presented on the viral surface and subject to various host immune pressures, which predisposes it to a greater range of genetic heterogeneity including insertions and deletions. Because the NP protein is highly abundant in virus infected cells, it provides an attractive target for the development of antigen-based serological assays. Taken together, this evidence provided rationale for using it as our antigen of choice for the iELISA, bELISA and FMIA.

In response to the recent outbreaks of highly virulent PEDV in North America (NA), PCR assays were quickly developed to detect the presence of PEDV RNA in intestine or fecal material. These assays provide an important tool in control of the virus; however, well-validated, high-throughput assays to detect antibodies following infection would provide additional valuable diagnostic tools for the swine industry. The ability to detect and evaluate antibody responses using serologic tests is important in efforts to answer basic production related questions. These questions may include whether a production site is naïve or has historically experienced a PEDV exposure, even though a producer has not seen obvious clinical signs; the level of immune response sows may have in relation to vaccination, initial wild-type virus infection or intentional feedback exposure; and whether sow immunity is inadequate when clinical infection occurs in individual litters after initial PEDV exposure in a herd.

One of the most pressing issues of PEDV disease is maintaining herd site biosecurity through exclusion measures to prevent viral entrance into swine units. However, PEDV infection may not always be obvious in finishing pigs so the widespread transport of these animals may represent additional risks. Thus, sensitive serological tests provide a valuable tool in the detection of recent infection to avoid the introduction of these animals into naïve herds.

Since PEDV was widespread in Europe in the 1970s and 1990s and more recently in Asia, various serologic tests have been developed and subjected to varying degrees of validation [14–20]. However, few assays have been developed using antigens associated with contemporary strains currently circulating across NA. The need to develop more sensitive serological assays has become paramount in order to address questions regarding PEDV infections and epidemiological transmission patterns, as well as to analyze disease progression.

Currently, serum virus neutralization (SVN) tests are the most widely employed serological assays used to detect PEDV antibodies. It is a test that is highly specific and useful for screening of antibody titer post vaccination [1, 16]. However, the test is expensive and labor intensive, requiring manual reading and interpretation of virus induced cytopathic effect (CPE) endpoints. Moreover, serum cytotoxicity can be mistaken for viral CPE, giving rise to false interpretations at lower serum dilutions.

Several laboratories have generated in-house indirect ELISAs using either virus derived antigen or recombinant structural proteins. Early indirect ELISAs were developed using Vero cell derived, whole virus preparations [15, 21] or Vero cell expressed viral proteins [16]. These methods may be problematic because serum from animals vaccinated with cell culture derived PEDV may cross-react with cellular components of ELISA antigen, causing low specificity and high background. Other groups have used recombinantly expressed, purified, structural S and NP proteins for iELISA serodiagnosis, but because low numbers of experimentally derived samples were used to evaluate the performance of the assay, full validation of the diagnostic sensitivities and specificities could not be assessed [19, 20].

Both the iELISA and bELISA formats have proven useful for the serodiagnosis of experimental and natural infections. Blocking or competitive ELISAs have been shown to be especially useful where a higher level of specificity is required. The increased specificity has been shown to be dependent on both the isotype and on the target specificity of the monoclonal or polyclonal antibodies [6, 17, 22]. Various laboratories have developed sensitive blocking ELISAs, and Carvajal et al. [17] demonstrated their bELISA was able to detect an antibody response 3 to 5 days earlier than IFA, which suggested higher sensitivity of the bELISA. In addition, the bELISA is valuable as a confirmatory test where unexpected positive results appear in presumably negative herds.

The fluorescent microsphere immunoassay is based on fluidic, particle array technology (Luminex Corp., Austin, TX) and has become increasingly standardized and accepted in applications involving the serologic diagnosis of autoimmune and animal infectious diseases [23, 24]. There are distinct advantages of the FMIA over the ELISA, which include higher sensitivity, higher sample throughput analysis, and the ability to multiplex and monitor exposure to multiple pathogens simultaneously in a single sample. In addition, multiple bead sets in the FMIA could be added to a standardized assay against newer virus subtypes that continue to emerge in the field or to assess antigenic/phylogenetic differences between genera of coronaviruses.

In this study, we report the adaptation of a recombinant, highly purified, NA PEDV-NP antigen to the development of iELISA, bELISA and FMIA platforms for the detection of PEDV antibodies in serum. These assays provide high throughput serological tests designed to address PEDV disease diagnostics. They were fully validated using a large number of serum samples of known status, and validation of the tests was detailed using methods for the validation of serological assays for the diagnosis of infectious diseases previously described by Jacobson for the Office International des Epizooties [25]. In addition, a fluorescent focus neutralization (FFN) assay was developed for the rapid evaluation of neutralizing antibody responses.

## Methods

### Ethics Statement

Procedures involving animals were approved by the South Dakota State University Institutional Animal Care and Use Committee (IACUC) under approval numbers 13-054A and 04-A034. Time course swine serum samples provided by Kansas State University were part of a separate PEDV challenge study conducted at the Biosecurity Research Institute approved by the Kansas State University Institutional Animal Care and Use Committee. All other serum samples were obtained as routine diagnostic sample submissions at the South Dakota ADRDL.

#### Animal samples for assay validation and time-course serological evaluation

For time course studies, serum samples from experimentally infected animals were obtained courtesy of Dr. Richard Hesse (Kansas State University Veterinary Diagnostic Laboratory, National Pork Board Grant #13-228). Thirty-three PEDV naïve 3-week-old feeder pigs were obtained from a private, high-health status swine production farm. . Of the 33 pigs, 23 were inoculated with PEDV at 4 weeks of age via intranasal and oral routes with a pool of gut derived intestinal contents that had been used as “feedback” inocula for controlled exposure of a sow herd. Serum was collected prior to challenge and at days 0, 6, 9, 14, 21, 28, 35 and 43 days post-infection (DPI). Multiple aliquots of all samples collected were shared with requesting laboratories to expand diagnostic testing and vaccine development capabilities.

To accurately assess the diagnostic sensitivity and specificity of the assays, samples of known serostatus for PEDV were used. This included sera from multiple animal populations including experimentally infected animals and serum samples from animals with known historical exposure to PEDV that were submitted to the South Dakota Animal Disease Research and Diagnostic Laboratory (ADRDL). PEDV negative sample sets included samples from PEDV negative control pigs used in experimental studies and selected high biosecurity herds with no history of PEDV. In addition, archived serum samples collected prior to the emergence of PEDV in the U.S., including samples testing positive for the related swine coronaviruses TGEV and PRCV (n= > 50), were used. The exact number of positive and negative sera used for sensitivity and specificity calculations per assay with statistical testing agreement calculations based on serum numbers is listed in Table 1. The majority of these sera were identical among assays, but limited serum volume did not allow for use of all sera samples among all assays.

**Table 1:** Evaluation of statistical agreement among serological testing platforms. Multiple comparison, inter-rater agreement (kappa association) was calculated among all four tests. Kappa values shown represent a statistical measure of test agreement and were calculated using MedCalc version 11.1.1.0

	FMIA	bELISA	Indirect ELISA	IFA
IFA	0.932	0.945	0.941	1
iELISA	0.919	0.923	1	0.941
bELISA	0.941	1	0.923	0.945
FMIA	1	0.941	0.919	0.932
Number Positive Serum Samples	158	158	158	158
Number Negative Serum Samples	361	361	361	361
Total Serum Samples Tested	519	519	519	519

#### Antigen production, expression of recombinant PEDV-NP protein

**Fig. 1:**
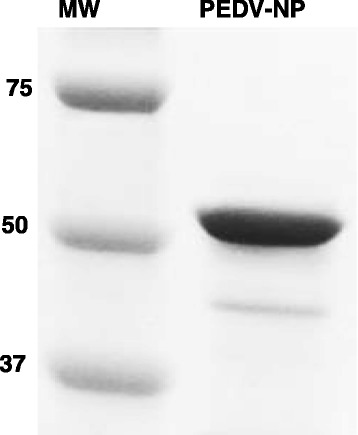
Purification of antibody capture antigen. SDS-PAGE/Coomassie blue staining of *E. coli* expressed and purified NA PEDV-NP antigen used to coat ELISA microtiter plates and FMIA microspheres. Molecular weight ladder (MW) PEDV-NP (51 kDa)

**Fig. 2:**
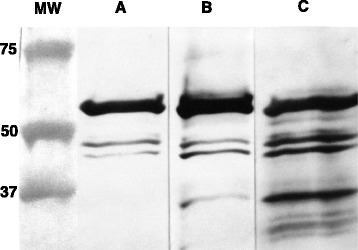
Antigen/antibody specificity. Western blot analysis showing detection of recombinant expressed NA PEDV-NP protein and specificity of the monoclonal antibody used in the bELISA. L- Molecular weight ladder. A- anti-PEDV-NP mAb 6–29. B- anti-polyhistidine mAb. C- anti PEDV-NP convalescent swine serum

The development and validation of the iELISA and bELISA made use of a recombinantly expressed full length NA PEDV-NP. The NP open reading frame (ORF) of PEDV was amplified from RNA extracted directly from intestinal contents by RT-PCR from a case submitted to the South Dakota ADRDL. It was subsequently directionally cloned into the *E. coli,* pET 28a(+), plasmid expression vector (Novagen, Madison, WI), then transformed into BL21-Codon Plus (DE3)-RP competent cells (Stratagene, La Jolla, CA) for protein expression. Primers used for the amplification of the full length (1323 bp) nucleoprotein were: PEDV-NP-fwd (5′-CGCGGATCCATGGCTTCTGTCAGTTTTCAG-3′); PEDV-NP-rev (5′- CACACTCGAGATTTCCTGTGTCGAAGATCTC-3′). Next, 20 μl of transformed cells were plated onto Luria-Bertani agar plates containing 50 μg of kanamycin/ml and incubated overnight. The following morning, colonies from the agar plates were added to 1 L of pre-warmed 2X yeast extract tryptone (YT) culture medium containing 50 μg kanamycin/ml and allowed to grow to an OD_600_ of 0.5 at 37 °C. PEDV-NP expression was induced using isopropyl β-D-1-thiogalactopyranoside (IPTG) at a final concentration of 1.0 mM to induce transcription of the Lac operon, and the *E. coli* was allowed to incubate for an additional 8 h at 37 °C with shaking at 200 RPM. The agar was strained out and bacteria pelleted by centrifugation at 12,000 g for 10 min at 4 °C. The pellet was resuspended in 40 ml of lysis buffer solution (B-PER, Pierce, Rockford, IL), incubated for 15 min at 20–22 °C, then centrifuged at 12,000 g to separate the soluble from insoluble proteins. The PEDV-NP recombinant protein was expressed as insoluble periplasmic inclusion bodies. The resulting 441 amino acid recombinant protein was denatured using 8 M urea, subsequently purified three times using nickel-NTA affinity column chromatography and refolded back to its native conformational state. Individual affinity column elutions were collected, pooled and confirmed by SDS-PAGE, then aliquoted/frozen at −80 °C. The correct nucleotide sequence was confirmed by sequence and restriction endonuclease analysis. The average protein yield produced by the pET28a-PEDV-NP plasmid construct was calculated to be 11 mg PEDV-NP per liter of 2XYT under the aforementioned conditions. The recombinant protein was detected and a predicted molecular weight of 51 kDa was confirmed via Western Blotting using convalescent sera, a 6X histidine-specific mAb (Novagen, Madison, WI) and a PEDV-NP specific mAb (Figs. 1 and 2).

#### mAb production and biotinylation

Two separate mAbs were developed in our laboratory (SD6-29 and SD17-103) that recognize both the native conformation of the PEDV-NP and the full length, linear, recombinant protein used in all antibody capture assays. Hybridomas were produced as previously described [26, 27]. Immunoglobulin isotyping of the resulting mAbs was performed using a commercial lateral flow assay (Serotec, Raleigh, NC). Subsequently, mouse ascites fluid was produced in pristane-primed mice, and the antibodies were purified and biotinylated for use as the detection moiety for the bELISA [23]. The conjugated antibody solution was quantified via the Lowry protein method, and carrier BSA was added to a final concentration of 10 mg/ml, then aliquoted and stored at −20 °C.

#### IFA

Vero-76 cells (ATCC CRL-1587) were cultured with MEM + 10 % FBS for 48–72 h until fully confluent in 96-well plates, then washed twice with MEM. Subsequently, alternating rows of 96-well plates were inoculated with the cell culture adapted PEDV NVSL-CO strain of PEDV (PEDV USA/Colorado/2013, GenBank accession number KF272920) at a multiplicity of infection (MOI) of 0.05 with MEM supplemented with 1.5 μg/ml trypsin (TPCK treated, bovine derived (Sigma, St. Louis, MO)). After incubation at 37 °C for 20–24 h, monolayers were fixed with 80 % acetone for 20 min. Serum samples were initially serially diluted from 1:40 to 1:320 with PBS in duplicate wells, and 100 μls of the diluted serum were added to each well. The plates were incubated at 37 °C for 1 h and then rinsed 3X with PBS. Next, fluorescein isothiocyanate (FITC)-conjugated goat anti-swine IgG (KPL, West Chester, PA) was prepared at a dilution of 1:15 with PBS and 50 μl added to each well. After 1 h of incubation at 37 °C, plates were rinsed 3X with PBS and examined using fluorescent microscopy. For each individual test, each PEDV infected well was compared to its respective uninfected partner well, and a positive sample was indicated if a PEDV specific fluorescent signal was observed at a serum dilution of 1:40 or greater. All samples were tested in duplicate, and the antibody titer was expressed as the mean of all replicates.

#### iELISA

The serological PEDV-NP indirect ELISA was performed by coating alternate wells of Immulon 1B, 96-well, microtiter plates (Thermo Labsystems, Franklin, MA) with 250 ng/well of purified, recombinantly expressed PEDV-NP antigen. The optimal dilution of the recombinant protein and secondary detection antibody was determined by a checkerboard titration that gave the highest signal to noise ratio. In addition, a single lot of pooled convalescent serum from PEDV infected pigs was used to generate quality control standards that gave high and low optical density (high OD = 2.0 to 2.5; low OD = 0.5 to 1.0; and negative OD < 0.2). PEDV-NP recombinant protein was diluted to 2.5 μg/ml in 15 mM sodium carbonate-35 mM sodium bicarbonate- antigen coating buffer (ACB) pH 9.6. Odd-numbered columns were coated with 100 μl of ACB plus antigen, while the even-numbered columns were coated with ACB without antigen, serving as background control. The plates were incubated for one hour at 37 °C, then washed 3X with PBS plus 0.05 % tween_20_ (PBST). Each well was then blocked with 200 μl of sample milk diluent (PBST plus 5 % nonfat dry milk, (SMD)) and allowed to incubate overnight at 4 °C. The following day, the plates were washed 3X with 300 μl of PBST. Test and control sera were diluted 1/50 in SMD, mixed, and 100 μl of the solution was added to each well. The plates were incubated for 1 h at 20–22 °C. Next, 100 μl of biotinylated, goat anti-swine detection antibody (Bethyl Laboratories, TX) was added at a concentration of 200 ng/ml of PBST and allowed to incubate at 20–22 °C for 1 h. The plate was washed 3X with 300 μl of PBST, then 100 μl of streptavidin-HRP conjugate (Pierce, Rockford, IL) was added and incubated for another hour at 20–22 °C, then washed and developed with 3,3′,5,5′- tetramethylbenzidine, peroxidase substrate (TMB) (Surmodics, Eden Prairie, MN). Color development progressed until the positive control attained a standard OD and was stopped using 2 N H_2_SO_4_. Colorimetric development was quantified spectrophotometrically at 450 nm with a ELx800 microplate reader (BioTek Instruments Inc., Winooski, VT) controlled by XCheck software (Idexx Laboratories, Westbrook, ME). The raw data was normalized and transformed into an Excel spreadsheet. Sample to Positive (S/P) ratios were calculated using the following formula: S/P = optical density (OD) of sample - OD of buffer/OD of positive control - OD of buffer.

#### bELISA

The serological bELISA was performed using Immulon 1B, 96-well microtiter plates (Thermo Labsystems, Franklin, MA). Alternate wells of each plate were coated with 500 ng per well of expressed PEDV-NP antigen. The optimal dilution of the recombinant protein and mAb antibody was determined by a checkerboard titration that gave the highest signal to noise ratio with an OD reading of approximately 2.0, in the absence of swine serum/competitor antibody. First, PEDV-NP recombinant protein was diluted to 2.5 μg/ml of ACB. Odd-numbered columns were coated with 100 μl of ACB plus antigen, while the even-numbered columns were coated with ACB without antigen serving as background control. The plates were incubated for 1 h at 37 °C, washed 3 times with PBST, then placed at 4 °C overnight. The following day, each well was blocked with 300 μl of SMD and incubated one hour at 37 °C. Plates were washed 3 times with PBST, and 100 μl of test and control sera were diluted 1/3 with PBST + 0.1 % nonfat dry milk and added to each of the duplicate wells. Plates were incubated 1 h at 37 °C. During sample incubation, PEDV-NP specific biotinylated, mAbs (SD6-29 and SD17-103) were adjusted to equal titers and mixed together in a 1:1 ratio. Next, 100 μl of a 1:40,000 dilution of the antibody detection mixture was added to the microtiter plate containing the competitive swine antibody, then swirled and incubated for an additional 30 min at 37 °C. The plates were washed 3 times, and 100 μl of high sensitivity, streptavidin-horseradish peroxidase conjugate (Pierce, Rockford, IL) was added to all wells of the microtiter plate for 1 h at 37 °C.

Plates were washed 4 times with PBST, and 100 μl of TMB was added to all wells and gently swirled. After approximately15 min, color development progressed until the negative control attained a standard OD of approximately 2.0 and was subsequently stopped using 2 N H_2_SO_4_. Colorimetric development was quantified spectrophotometrically at 450 nm with an ELx800 microplate reader (BioTek Instruments Inc., Winooski, VT) controlled by XCheck software (Idexx Laboratories, Westbrook, ME). The raw data was normalized and transformed into a Excel spreadsheet, and the percent inhibition (PI) ratio was calculated using the following formula: PI = 1-{(OD of sample - OD of buffer)/(OD of negative control – OD of buffer)} X 100.

#### Preparation of PEDV-NP coupled microspheres for the FMIA

A two-step carbodiimide coupling procedure was used to couple NA PEDV-NP protein to Luminex™ microspheres. Briefly, the coupling of fluorescent microsphere was performed by washing 3.125 × 10^6^ microspheres twice with 250 μl activation buffer (0.1MNaH_2_PO_4_, pH6.2) and sonicating them for 60 s after each wash. Microspheres were activated for 20 min at 20–22 °C in 500 μl activation buffer containing 2.5 mg *N*-hydroxysulfosuccinimide (sulfo-NHS) and 2.5 mg *N*-(3- dimethylaminopropyl)-*N*-ethylcarbodiimide (EDC) (Pierce Chemical, Rockford, IL). Activated microspheres were washed twice with PBS and sonicated. Coupling was initiated by the addition of 12.5 μg of recombinant NA PEDV-NP protein, brought to a final volume of 500 μl with PBS and incubated in the dark for 3 h at 20–22 °C with rotation. Coupled microspheres were washed once with 1 ml of PBS plus 0.05 % NaN_3_ and 1.0 % bovine serum albumin (PBS-NB). Next, the microspheres were blocked with 1 ml of PBS-NB for 30 min to reduce nonspecific binding. Microspheres were then washed twice with PBS-NB and resuspended in PBS-NB to a final concentration of 2.0 × 10^6^ antigen-coupled microspheres/ml.

#### FMIA

A 96-well hydrophilic membrane filter plate was blocked for 2 min with 150 μl of PBS-NB, and then the liquid was aspirated via vacuum manifold. The plates were wetted with 20 μl of PBS-NB buffer to prevent drying. Next, 50 μl of serum (diluted 1:50 in PBS-NB) was added to duplicate wells of the filter plate along with 50 μl of PBS-NB containing 2.5 × 10^3^ antigen-coupled microspheres. Since the microspheres and reporter moieties are light sensitive, all incubations were performed in the dark by sealing the plate with foil. Subsequently, the FMIA plate was incubated at 20–22 °C for 1 h on a plate shaker rotating at 600 rpm. The plate was washed 3 times with 200 μl of PBST. Next, 50 μl of anti-swine, biotinylated IgA (heavy & light chain, diluted in PBS-NB; Bethyl Laboratories) or IgG-FC specific polyclonal antibodies (diluted 1:2,000 dilution in PBS-NB; Bethyl Laboratories) were added to the filter plate and incubated at 20–22 °C for 1 h. NP IgM and IgG isotype-specific antibody levels were detected using PEDV-NP coated microspheres, but speciated by means of individual and separate IgM and IgG-specific secondary antibodies. Since validation was performed using serum, and because IgA is present in very low amounts, IgA specific secondary antibodies were not used at this step. After incubation with the secondary antibodies, 50 μl of streptavidin phycoerythrin (2.5 μg/ml in PBS-NB, Molecular Probes) was added to each well and incubated for 30 min at 20–22 °C with shaking. The supernatant was aspirated, and the plate was washed 3 times with PBST. Finally, the microspheres were resuspended in 125 μl of PBST per well and transferred to a clear 96-well polystyrene optical plate. Coupled microspheres were analyzed through a dual-laser Bio-Rad Bio-Plex 200 instrument. The median fluorescent intensity (MFI) for 100 microspheres corresponding to each individual bead analyte was recorded for each well. All reported MFI measurements were normalized via F - F_0_, where F_0_ was the background signal determined from the fluorescence measurement of a test sample in uncoated beads and F was the MFI for a serological test sample using antigen-coated beads.

#### Establishment of serological reference standards for ELISA and FMIA development

Four serological reference serum sets were constructed as standards termed high, medium, low and negative to serve as internal quality control standards and to mathematically normalize individual samples for objective comparisons between testing platforms. The high-labeled standard was designed to generate an OD above 2.0 for the iELISA and bELISA and an MFI of approximately 25,000 for the FMIA. The high standard was used exclusively for the mathematical determination of the serological response (S/P ratio) of samples used for test validation. The medium standard generated a response of between 1.5 and 2.0 OD for the two ELISAs and approximately 10,000 MFI for the FMIA. The low standard was designed to deliver a signal slightly above threshold level for all 3 tests, and the negative serum generated an OD or MFI to a background level of less than 0.2 OD for the ELISAs and 600 MFI for the FMIA.

#### Validation methods for the determination of diagnostic sensitivity, specificity, repeatability and threshold cutoff level

To accurately assess the diagnostic sensitivity and specificity of the assays, samples of known serostatus for PEDV were used. This included sera from multiple animal populations including experimentally infected animals and serum samples submitted to the South Dakota ADRDL. PEDV negative sample sets included samples from selected high biosecurity herds with no history of PEDV and archived serum samples collected prior to the emergence of PEDV in the U.S., including samples testing positive for the related swine coronaviruses TGEV and PRCV. Known positive samples were collected from pigs that were naturally infected at least 3 weeks prior to collection and were previously positive by PCR. The negative-testing sample population (uninfected animals) consisted of maximally 980 PEDV negative serum samples, while the positive-testing (infected) population was composed of 516 serum samples. Receiver operating characteristic (ROC) analysis was calculated for each assay to assess diagnostic performance, which included determination of sensitivity, specificity and threshold cutoff using MedCalc version 11.1.1.0 (MedCalc software, Mariakerke, Belgium).

The repeatability of each assay was assessed by running the same internal quality control serum standards in multiple replicates within the same run or between runs. For the iELISA and the bELISA, the intra-assay repeatability was calculated for 48 replicates on 3 separate plates, then repeated over a 3-day period for inter-assay repeatability assessment. The values for each assay were expressed as a mean, standard deviation and percent coefficient of variation (CV%) for repeated measure.

#### Measurement of statistical testing agreement

Multiple comparison, inter-rater agreement (kappa measure of association) was calculated among all four tests (bELISA, iELISA, FMIA and IFA) using IBM, SPSS version 20 software (SPSS Inc., Chicago, IL). The sample cohort used was a well-characterized set of serum samples collected from “positive testing” experimentally infected pigs over time courtesy of Dr. Richard Hesse (n = 158) and from archived experimental control uninfected PEDV “negative testing” animals. The interpretation of kappa can be rated as follows: Kappa less than 0.0, “poor” agreement; between 0.0 and 0.20, “slight” agreement; between 0.21 and 0.40, “fair” agreement; between 0.41 and 0.60, “moderate” agreement; between 0.61 and 0.80, “substantial” agreement; and between 0.81 and 1.0, “almost perfect” agreement [28, 29].

#### FFN

A PEDV virus neutralization assay using a FFN format was developed for rapid detection of neutralizing antibodies produced in response to PEDV infection. The FFN was evaluated using serum samples or rennet treated milk and colostrum samples. Heat-inactivated samples were diluted in a 2-fold dilution series starting at 1:10 in MEM plus 1.5 μg/ml TPCK-treated trypsin in 96-well plates. An equal amount of cell culture adapted PEDV stock at a concentration of 100 foci forming units/100 μl was added to each well and plates incubated for 1 h at 37 °C. The virus/sample mixture was then added to washed confluent monolayers of Vero-76 cells and incubated for 2 h at 37 °C. Plates were washed again with MEM/TPCK-trypsin medium and incubated 20–24 h to allow for replication of non-neutralized virus. Plates were then fixed with 80 % acetone and stained with FITC conjugated mAb SD6-29 to allow visualization of infected cells. Endpoint neutralization titers were determined as the highest serum, milk or colostrum dilution resulting in a 90 % or greater reduction in fluorescent foci relative to controls.

## Results

### Expression of recombinant PEDV-NP antigen

As shown in Fig. 1, the purity of the recombinant protein was assessed via SDS-PAGE and gave a band that migrated corresponding to the expected molecular mass of 51 kDa upon staining with Coomassie brilliant blue R250. The protein yield of the IPTG induced *E. coli* culture was calculated to be approximately 11 mg PEDV-NP/liter of 2XYT medium with a purity of greater than 95 %. The identity of the protein was further characterized by Western blot using convalescent swine serum, an anti-His mAb and an anti-PEDV-NP mAb (Fig. 2).

### Diagnostic sensitivity, specificity, repeatability and threshold cutoff level

**Fig. 3:**
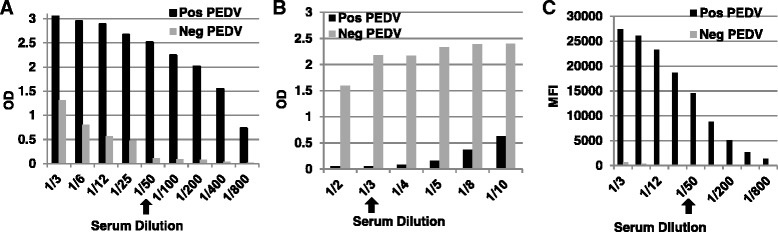
Serum dilution optimization for both ELISA assays and FMIA. Reference serum standard was titrated 2-fold in antigen coated wells at a fixed concentration in order to gauge a maximum signal-to-noise ratio for each assay **a** iELISA, **b** bELISA, **c** FMIA. Arrows show the optimum dilution of swine serum from which the highest signal to noise ratio was achieved

To optimize the serologic assays, various antigen and serum dilutions were used to determine optimum concentrations. All 3 tests were optimized in a checkerboard fashion to maximize signal-to-noise ratios. It was determined by antigen titration that the optimal coating of Luminex™/FMIA microspheres was achieved at a concentration of 12.5 μg protein per 3.125 × 10^6^ microspheres. Similarly, the optimum coating of both the iELISA and bELISA plates was achieved at a concentration of 250 ng/well. In addition, to determine the optimum serum dilution for each of the testing platforms, a well-characterized PEDV “high” positive serum standard was serially diluted in a log_2_ titration against antigen coated microspheres (FMIA) or antigen coated ELISA wells at a fixed concentration. Figure 3 shows concentration-dependent OD or MFI signals of various serum standards. Overall, sample absorbance increased inversely proportional to the serum dilution. However, based upon the highest signal-to-noise ratio, it was determined that the optimal serum dilution for the bELISA was 1/3, while the iELISA and FMIA each demonstrated an optimum dilution of 1/50 as indicated by arrows (Fig. 3).

ROC analysis to determine sensitivity, specificity and threshold cut-off levels was performed using large numbers of swine serum samples and demonstrated excellent agreement (>0.91 kappa scores) between assays with good intra and inter assay repeatability (Table 1). None of the known positive TGEV or PRCV samples tested was shown to cross-react.

**Fig. 4:**
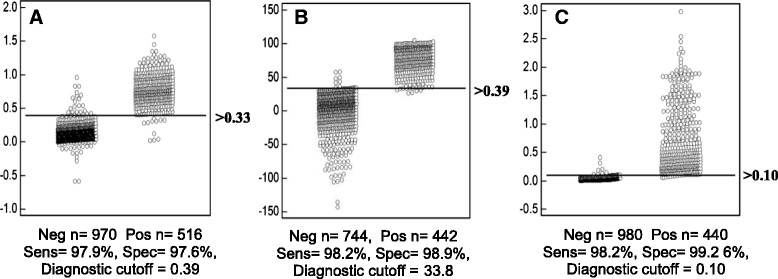
Receiver operator characteristic (ROC) validation and determination of diagnostic sensitivity and specificity of the PEDV-NP iELISA, bELISA and FMIA assays. Diagnostic sensitivity and specificity were calculated using serum samples from a known PED-uninfected and PED-infected population. ROC analysis was performed using MedCalc version 11.1.1.0 (MedCalc software, Mariakerke, Belgium). In each panel, the dot plot on the left represents the negative testing population, and the dot plot on the right represents the positive population. The horizontal line bisecting the dot plots represents the cutoff value that gives the optimal diagnostic sensitivity and specificity. **a** Serum iELISA, **b** Serum bELISA, **c **Serum FMIA

The optimal cutoff values and corresponding sensitivity and specificity of each individual test are presented in Fig. 4. Specifically, ROC analysis for the iELISA and bELISA showed similar sensitivity and specificity of 97.9 and 97.6 %; and 98.2 and 98.9 %, respectively. The ROC analysis for the FMIA showed estimated sensitivity and specificity of 98.2 and 99.2 %, respectively. Although the FMIA showed an identical sensitivity as the bELISA, it demonstrated the highest degree of specificity of all three assays at 99.2 %. This observation was not surprising given that FMIA technology inherently imparts greater sensitivity and a larger dynamic range than the ELISA platform [30].

In addition to determining cutoff values, sensitivities and specificities, multiple comparison tests were performed to calculate the degree of agreement among the ELISA, FMIA and IFA tests. Specifically, the Kappa test demonstrated all diagnostic platforms had kappa values greater than 0.91, which demonstrates that all 4 tests are in “almost perfect” agreement with each other.

### Assessment of repeatability

The iELISA and bELISA demonstrated slightly lower %CVs than the FMIA with 3.7 %, 6.8 %, 10.7 % intra-assay variability for bELISA, iELISA and FMIA respectively. Inter-assay %CVs were 5.0, 5.6 and 7.7 % for the bELISA, iELISA and FMIA respectively. Nonetheless, all the CVs were 10.7 % or less, which demonstrated that the tests were highly repeatable in a diagnostic application.

### Evaluation of a kinetic PEDV antibody response

**Fig. 5:**
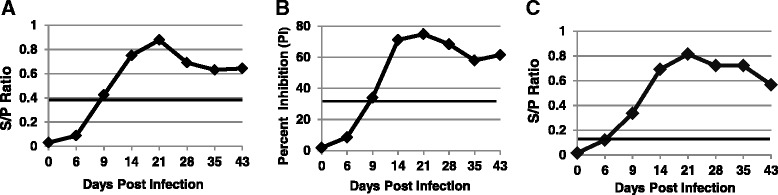
Kinetic time course antibody evaluation. Antibody time course kinetics were calculated for each of the ELISAs and FMIA using serum samples from experimentally infected pigs collected at weekly intervals. The horizontal line indicates the diagnostic cutoff for each test. All three tests demonstrate similar kinetic curve responses via their calculated S/P values. **a** Antibody kinetic time course via iELISA, **b** Antibody kinetic time course via bELISA, **c** Antibody kinetic time course via FMIA

As shown in Fig. 5, a mean antibody response to PEDV-NP could be detected as early as 9 DPI for both the iELISA and bELISA. The FMIA detected PEDV-NP antibodies slightly earlier at 6 DPI. All 3 tests detected the duration of antibody out to the 43 DPI time-point in this study but demonstrated a decline in detectable antibody after 21 DPI.

### Application of the FMIA to isotype PEDV-NP specific antibodies

**Fig. 6:**
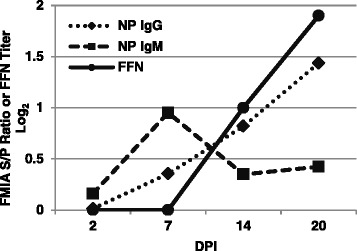
FFN antibody and FMIA isotype time course evaluation. Using serum collected over time from experimentally infected pigs, the FMIA demonstrates the kinetic nucleoprotein-directed, isotype-specific response of IgG and IgM in serum. In addition, the data show a concomitant appearance of neutralizing antibodies as soon as 14 DPI

High levels of PEDV-NP specific IgM antibodies were observed at 7 DPI compared to IgG (Fig. 6). However, the IgM antibodies decreased to barely detectable levels by 20 DPI. IgG continued to increase linearly to 20 DPI. There is a concomitant appearance of neutralizing antibodies by 14 DPI.

### FFN

**Fig. 7:**
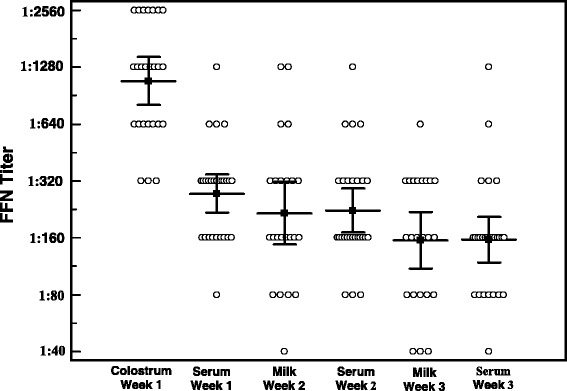
Assessment of neutralizing antibody titers in different sample matrices following PEDV exposure. FFN titers were detected in various sample matrices including colostrum (n = 25), milk (n = 23) and serum (n = 27) collected at the time of farrowing and weekly for two weeks post-farrowing. Error bars indicate a 95 % confident interval for mean titers indicated by horizontal lines

The FFN assay was initially evaluated using sequential serum samples from experimentally inoculated piglets. Additional evaluation was conducted using 250 serum samples from known PEDV naïve herds and 250 samples from herds with documented PEDV exposure, collected at least 3 weeks after initial PCR diagnosis and whole herd feedback. Experimentally inoculated piglets demonstrated detectable seroconversion by 14 DPI (Fig. 6). Essentially all samples from PEDV naïve animals had serum FFN endpoint titers of <1:20 while most samples from the PEDV positive set had endpoint titers ranging from 1:40 to 1:1280 (data not shown). Further evaluation of the FFN included serum, milk and colostrum samples from 27 sows from a herd that had experienced an acute PEDV outbreak 6 to 7 weeks prior to farrowing. All animals were exposed to live virus twice within the first week of the outbreak, followed by one dose of Harrisvaccines Porcine Epidemic Diarrhea Vaccine, RNA (Harrisvaccines, Inc., Ames, IA) at 1 week pre-farrow. Serum and colostrum samples were tested at the time of farrowing, followed by serum and milk samples at 1 week and 2 weeks later. As shown in Fig. 7, mean colostrum titers were approximately 4-fold higher than serum titers at the time of farrowing. At later time-points, serum and milk titers were similar in magnitude, although substantial animal to animal variation was apparent.

## Discussion

Overall, this repertoire of assays is useful for initial identification and efficient, high throughput quantitation of PEDV antibodies. We evaluated all three diagnostic platforms against a well characterized IFA and compared the individual serum IgM and IgG kinetic antibody responses in an FMIA to the appearance of neutralizing antibody as detected by the FFN assay. Each of the antibody-capture assays was validated using a large number of serum samples (n >1100) based upon the assay validation methods of Jacobson, which is supported by the Office International des Epizooties [25].

Since PEDV was first identified in the U.S. in May 2013, it has spread rapidly to at least 33 states (www.aasv.org) and has been reported in Mexico and Canada [31]. The virus causes severe gastroenteritis, destroying villus enterocytes in pigs of all ages, and is characterized by vomiting and diarrhea, leading to subsequent dehydration, high mortality rates and economic losses, particularly in nursery piglets [3, 32]. A variety of serological tests have been developed against PEDV, but they vary by antigen used and in the degree of validation. In addition, few have used North American NP based antigens or compared the array of serologic assays described here. In the current study, four tests (IFA, bELISA, iELISA, FMIA) showed strong correlation. Each has advantages, which dictate how they will be used in the field. In addition, newly developed NP mAbs were used in the bELISA and for expediting FFN testing in the detection of neutralizing antibodies.

In the development of the ELISAs and FMIA, the full length NA PEDV-NP gene was amplified directly from RNA extracted from PEDV-infected ileal tissue. Multiple sequence alignment analysis showed that the amplified NP gene shared a 100 % nucleotide homology with that of the US Colorado strain isolated in 2013 (Genbank accession no. 13–019349). Several authors confirm that the NP carries multiple antigenic determinants that are conserved among the *Coronaviridae* [33, 34]. However, we performed one-way cross-reactivity testing using serum from TGEV and PRCV, and no antibody cross-reactivity was detected within any of our assays. In addition to being highly conserved among various PEDV variants, the NP is the most abundant viral protein expressed in PEDV infected cells, making it an attractive target antigen [12, 13]. Using Western blotting experiments, we confirmed the finding of Hou et al. [19], in which they observed the level of expression of NP protein to be significantly higher than the level of the spike protein. Our study demonstrated that it is possible to achieve a protein yield of over 10 mg per liter of culture with a purity of greater than 95 %.

The recombinant NP has previously been identified as a useful antigen in other ELISAs developed to detect antibodies in pigs located in China and Korea [19]. In a study by Hou et al. [19], the authors showed similar sensitivities and specificities of their iELISA compared to the iELISA described in this study. However, smaller numbers of known positive and negative samples were evaluated than in the current study.

Since no test has 100 % specificity, a bELISA was developed that is useful for confirmatory testing due to its higher inherent specificity than the iELISA [35]. Blocking or competitive ELISAs have been constructed using monoclonal antibodies in PEDV serodiagnosis, and the specific methodology can affect the overall specificity and performance of the assay. Our method was based upon coating plates with highly purified NA PEDV-NP, then using a combination of two separate NA, anti- PEDV-NP specific, biotinylated, monoclonal antibodies as the blocking/competitive detection step. This allows the capture of anti-NP antibodies at higher quantities and those with a greater range of antigen specificities. The analytical specificity of the NP-based bELISA is also dependent on the affinity of the mAbs used. The antibodies used in this study are directed against conserved epitopes on the nucleocapsid protein without any evidence of cross-reactivity to any other genera of *Alphacoronavirus* tested. A previous assessment of antigenic cross-reactivity was performed using these same mAbs against different strains of PEDV and TGEV [7]. In that study, the authors reported that both mAbs used in the bELISA reacted with all PEDV strains tested, namely the homologous US isolate PC22A and the heterologous strains S INDEL IOWA 106, S 197DEL PC177 and CV777, at similar titers. Neither of the PEDV-NP mAbs cross-reacted with either the TGEV Miller or Purdue strains. Not only were the bELISA mAbs tested for heterologous cross-reactivity, but all three diagnostic platforms were evaluated in their ability to capture antibody against TGEV and PRCV, and there was no cross-reactivity to either heterologous virus.

Serology testing with IFA, iELISA, bELISA or FMIA is useful in determining whether pigs were previously infected with PEDV, or if piglets have acquired antibodies through colostrum (eg. passive antibody transfer). However, tests that evaluate the functionality of the antibodies such as the FFN are needed to determine if the detected immune response could be helpful in providing protection to nursing piglets. Neutralizing antibodies may be protective through actions including blocking uptake of the virus into cells, preventing virus binding to receptors on cells, preventing uncoating of the virus genomes in endosomes and/or causing aggregation of virus particles. For enveloped viruses, such as PEDV, lysis of the virus may also occur when antiviral antibodies and serum complement disrupt the viral membrane. For these reasons, an FFN-based virus neutralization assay was developed to assess levels of PEDV neutralizing antibodies in serum, milk or colostrum samples. The FFN provides a more rapid determination of neutralizing antibody levels than is possible with traditional virus neutralization assays that rely on visualization of virus-induced CPE after three or more days incubation to allow for full development of PEDV CPE. The direct observation of fluorescent stained infected cells, or lack of stained infected cells in the case of virus neutralization, allows for simple endpoint determination. This feature is particularly valuable when dealing with a fastidious, trypsin-dependent virus such as PEDV where CPE-based endpoints may not be obvious or may be confused with trypsin-induced CPE in the cell monolayer. Although neutralizing antibodies present in the serum would not be expected to provide direct protection from a strictly enteric infection such as PEDV, our data suggest a correlation between detectable neutralizing antibody levels in the serum and those present in milk and colostrum of previously exposed or vaccinated sows.

Some correlation between PEDV neutralization results and ELISA results exists as described in the literature. One study performed a comparative analysis between a whole-virus antigen ELISA and a serum neutralization test for the serodiagnosis of PEDV [21]. The presence of antibodies was confirmed by each test, and an overall testing agreement of 84.2 % was demonstrated using 1024 field serum samples. Furthermore, a pairwise correlation was performed that showed corrected cutoff values between the ELISA OD and SN titers having an R value of 0.837, indicating that the CPE-based neutralization test had roughly the same reliability as the ELISA test [21].

Newer technologies such as the FMIA are useful for the detection of antibodies against multiple antigens simultaneously for surveillance purposes. FMIA are bead based assays for simultaneous high throughput detection of antibodies to multiple antigens. The FMIA differs from the ELISA since it involves a fluid incubation step with “beads suspended in solution, which allows for higher surface area exposure in 3 dimensions” [30]. Therefore, there is a shorter diffusion path to antibody binding sites on the antigen coated beads resulting in rapid reaction times. Instead of a method using an enzymatic reaction such as with the ELISA, the FMIA detection is with laser technology, which results in a shorter detection time. This PEDV antigen specific bead set can be “mixed” with additional coated beads to other antigens, such as SIV, PCV2, PRRSV or other pathogens, for simultaneous detection of antibodies to these antigens. In addition, an FMIA could be developed for differentiation of wild-type infected vs vaccinated animals (DIVA) if proteins used in the vaccine were different from those produced in a wild-type infection.

Individual kinetic serum IgG and IgM levels were measured by FMIA in experimentally infected animals over time. The appearance of the IgM subclass is considered an immunological parameter of early infection and generally appears prior to the appearance of IgG, and this was confirmed in our study. This was in contrast to the data of Woo et al. [36], which was unable to detect IgM antibodies using their NP-based indirect ELISA. IgG antibodies may be more easily detected as they are characterized by higher antigen affinity but lower avidity than IgM [37]. Further understanding of various antibody profiles will provide important information on the ability of vaccines to stimulate a protective immune response.

## Conclusions

These well-validated NA PEDV iELISA, bELISA, FMIA and FFN assays are useful for a range of serological investigations. They can serve as a complement to nucleic acid detection and determine the PEDV status of asymptomatic individuals for cost-effective tools in management strategies and monitoring virus exposure within the herd. The FMIA will be useful for isotyping the antibody responses and in multiplexing for determining exposure to multiple pathogens simultaneously. In addition, the FFN is useful for determining whether the antibodies measured are providing a biological function of blocking virus infectivity. Work is ongoing to further validate these assays on other sample matrices such as milk and colostrum for measuring passive transfer of antibodies and oral fluids for pen-based surveillance.

## Abbreviations

PEDV: Porcine epidemic diarrhea virus
IFA: Immunofluorescent assay
iELISA: Indirect enzyme linked immunosorbent assay
bELISA: Blocking enzyme linked immunosorbent assay
FMIA: Fluorescent microsphere immunoassay
FFN: Fluorescent Focus Neutralization
ORF: Open reading frame
UTR: Untranslated region
NP: Nucleoprotein
S: Spike
E: Envelope
mAb: Monoclonal antibody
ROC: Receiver operator characteristic
TGEV: Transmissible gastroenteritis virus
PRCV: Porcine respiratory coronavirus
NA: North American
CPE: Cytopathic effect
DPI: Days post-infection
ADRDL: Animal Disease Research and Diagnostic Laboratory
RT-PCR: Reverse transcriptase polymerase chain reaction
OD: Optical density
IPTG: Isopropyl β-D-1-thiogalactopyranoside
SDS-PAGE: Sodium dodecyl sulfate-polyacrylamide gel electrophoresis
IACUC: Institutional Animal Care and Use Committee
HAT medium: Hypoxanthine-aminopterin-thymidine medium
DMSO: Dimethyl sulfoxide
BSA: Bovine serum albumin
MEM: Modified Eagles Medium
FBS: Fetal bovine serum
NVSL: National Veterinary Services Laboratories
MOI: Multiplicity of infection
TPCK: L-1-Tosylamide-2-phenylethyl chloromethyl ketone
FITC: Fluorescein isothiocyanate
ACB: Antigen coating buffer
SMD: Sample milk diluent
HRP: Horseradish peroxidase
TMB: 3,3′,5,5′- tetramethylbenzidine
PI: Percent inhibition
MFI: Median fluorescent intensity
SIV: Swine influenza virus
PCV-2: Porcine circovirus type 2
PRRSV: Porcine reproductive and respiratory syndrome virus
